# Efficacy of a Muscarinic (M1) Positive Allosteric Modulator, SUVN‐I7016031 in Animal Models of Dementia

**DOI:** 10.1002/alz.087197

**Published:** 2025-01-09

**Authors:** Renny Abraham, Rajesh Babu Medapati, Surendra Petlu, Ramkumar Subramanian, Rajesh Kumar Badange, Kumar Bojja, Anil Shinde, Parusharamulu Molgara, Ramakrishna Nirogi

**Affiliations:** ^1^ Suven Life Sciences, Hyderabad, Telangana India; ^2^ Suven Life Sciences, Hyderabad India

## Abstract

**Background:**

SUVN‐I7016031 is a novel and selective positive allosteric modulator (PAM) of the M1 subtype of the muscarinic acetylcholine receptors (mAChRs). The proposed primary indication for SUVN‐I7016031 is in the treatment of dementia such as Alzheimer’s disease dementia (ADD) and Parkinson’s disease dementia (PDD). In the current research, the pharmacological properties of SUVN‐I7016031 in various types of dementia were investigated.

**Method:**

SUVN‐I7016301 was characterized using a calcium mobilization assay. The binding affinity towards the orthosteric M1 ‐ M5 site was investigated. The effect of SUVN‐I7016031 on neuronal spike rate in coronal hippocampal slice electrophysiology was studied in agonist and PAM modes of testing. The pharmacokinetic properties of SUVN‐I7016031 were studied both in rodent and non‐rodent species. The effect of SUVN‐I7016031 on MK‐801 induced memory deficits in rats using object recognition task (ORT) and on time‐induced social memory deficits in rats using social recognition task (SRT) was studied. The efficacy of SUVN‐I7016031 in a rat model of PDD was investigated. The effects of SUVN‐I7016031 (10‐60 mg/kg, *p.o*.) on inositol 1 phosphate (IP‐1) levels were studied in rats.

**Result:**

Functionally, SUVN‐I7016031 was found to be a positive allosteric modulator at the M1 receptor with an allosteric potency EC_50_ of 355 nM. In hippocampal slice electrophysiology studies, the EC_50_ and EC_30_ values were ∼527 nM and ∼326 nM, respectively. No significant binding towards the orthosteric site at the muscarinic M1 to M5 receptor was observed. SUVN‐I7016031 showed good oral bioavailability in rats, dogs, and monkeys. SUVN‐I7016031 was found to have brain penetration properties with adequate free fraction. SUVN‐I7016031 reversed delay‐induced memory disruption in adult rats in a SRT and antagonized MK‐801 induced memory disruption in ORT. SUVN‐I7016031 was found to reverse haloperidol induced memory deficits, a rat model of PDD. Treatment with SUVN‐I7016031 produced a significant increase in striatal inositol 1‐phosphate (IP‐1) levels in rats, providing in‐vivo support for the activation of M1 mAChRs by SUVN‐I7016031.

**Conclusion:**

SUVN‐I7016031 is a novel, potent, and selective M1‐PAM that demonstrated pro‐cognitive effects in animal models of PDD and ADD.

